# Environmental enrichment improves the growth rate, behavioral and physiological response of juveniles of *Clarias gariepinus* under laboratory conditions

**DOI:** 10.3389/fvets.2022.980364

**Published:** 2022-10-12

**Authors:** Oluwaseun Christianah Ojelade, Samuel Olutunde Durosaro, Abiodun O. Akinde, Ikililu Abdulraheem, Mathew B. Oladepo, Comfort A. Sopein, Abiodun S. Bhadmus, Mary Olateju

**Affiliations:** ^1^Department of Aquaculture and Fisheries Management, Federal University of Agriculture, Abeokuta, Ogun, Nigeria; ^2^Department of Animal Breeding and Genetics, Federal University of Agriculture, Abeokuta, Ogun, Nigeria

**Keywords:** aggressive, African catfish, enrichment, fish welfare, stress in fish

## Abstract

Environmental enrichment (EE) improves the growth rate and welfare of some cultured fishes. However, most cultured fish species are raised in non-enriched housing conditions. *Clarias gariepinus* is an important commercial fish species, but little is known about the effect of EE on their welfare. This study examined the effect of different EE on the survival rate (SR), growth [mean weight gain (MWG), specific growth rate (SGR) and feed conversion ratio (FCR)], behavioral (feed response, aggressive acts and shoaling time) and physiological responses (blood glucose) of *C. gariepinus. One hundred* and twenty juveniles of *C. gariepinus* (31.65 ± 0.69 g) were randomly allocated at 10 fish/tank and subjected to either Plant Enriched (PE), Substratum Enriched (SE), Plant and Substratum Enriched (PSE) and Non-Enriched (NE) tanks in triplicates for 56-days. Behavioral acts were observed for 10 min twice daily, and glucose level in blood samples was evaluated. Data were checked for normality using the Shapiro-Wilk test before being analyzed with the Kruskal-Wallis test. SR and MWG were significantly higher in *Clarias gariepinus* exposed to SE, with no significant differences among PE, PSE and NE treatments. There was no significant difference between the SGR of PSE and NE. FCR was similar between treatments. The highest condition factor (k) was recorded in SE tanks. Duration of feed response was shorter in SE, but there was no significant difference between the feed response of *C. gariepinus* exposed to PE and PSE. *C. gariepinus* exposed to PE, SE and PSE displayed a similar frequency of aggressive acts. African catfish reared in NE (barren) tanks had the least duration of shoaling period. The experiment consistently found the highest and least glucose values in PSE and SE. In conclusion, environmentally enriched housing tanks with SE resulted in the best MWG with a reduced level of aggression in *C. gariepinus* under laboratory conditions. Thus, EE might be applicable to boost fish productivity on a commercial scale.

## Introduction

The aquaculture sector makes a tremendous global contribution to the development of a nation in terms of provision of employment, fish food security, nutritional diet and a trade commodity for export (1). The aquaculture industry has been the fastest-growing global agro-industrial sector in the last four decades (2, 3). FAO Organization (4) and Franks and Ewell (5) reported a total of 82.12 million metric tons of farmed aquatic animals, which constitute around 250–408 billion fish species for the rising global human population. Furthermore, the aquaculture industry offers a great potential for boosting fish production at a rate that can outpace the rising domestic demand if the welfare of the cultured fish species is improved (3, 6, 7). However, most conventional rearing environments for fish culture are mostly barren. They lack a physical form of improvement or enrichments that could aid natural behavior in cultured fish to promote an optimum growth rate and welfare (8, 9). Interestingly, improving the rearing environment of cultured fish species is welfare friendly (6, 10, 11) and can serve as a growth booster (9, 12); thus, applying environmental enrichment could improve the production rate of *Clarias gariepinus* species for sustainability.

*Clarias gariepinus* (African catfish) is Africa's most popularly cultured finfish by Aquaculturists (2, 13). The fish is highly preferred for its ease of culture, general acceptability and high economic value (14). Furthermore, fish farmers mostly prefer the species due to its resistance to diseases, hardiness and fast growth rate (15). African catfish can tolerate a wide range of freshwater habitats and can still survive for weeks when they burrow into the sediment and mud of ponds. Consequently, *C. gariepinus* (African catfish) is well-studied in terms of nutrition (16, 17), feeding behavior (18, 19), management and reproduction techniques (20) and welfare (21–23), among others. In addition, Hossain and Beveridge (24) studied the effect of light and shelter on the growth and survival of *C. gariepinus*, Schram et al. (25) enriched the diet of *C. gariepinus* with functional selenium, Arechavala-Lopez et al. (26) reviewed the effect of environmental enrichment on cultured fish species and (22) assessed the effect of chronic stressor on welfare indicators of *C. gariepinus*. However, to the best of our knowledge, there is a paucity of information on the effect of environmental enrichment on growth indices, behavioral and the general wellbeing of this important tropical fish species, which calls for urgent attention to improve fish production efficiency and welfare.

Animal welfare can be described as the feelings experienced by animals, i.e., the presence of positive feelings or pleasure and the absence of strong negative feelings or suffering in the rearing environment of the animal (27, 28). Interestingly, animal welfare protections have been established for a variety of farmed species in developed countries (29, 30), yet the concept of fish welfare is gaining increasing public interest in developing countries. In most cases, fish are often categorized as aquatic animals, and their welfare is most often ignored in animal welfare decision-making policies. However, Sneddon et al. (31) and Brown (32) described fish as sentient beings that can experience good or bad feelings, pain or emotional states. In addition, Mason and Lavery (33) reviewed the uncertainty of the sentience nature of fish and opined that it is imperative to protect the welfare of fish and treat them as sentient animals. Consequently, the welfare of fish species must be given utmost attention to develop the aquaculture sector for sustainability. In the same vein, the utilization of different forms of environmental enrichment to improve the welfare of cultured fishes has remained an important global issue that is mostly being pursued by researchers, animal rights organizations and many producers to improve the productivity and welfare of fish species toward sustainability (26, 34–37). However, until now, environmental enrichment has not been applied to improve the welfare of tropical aquatic animals such as African catfish.

Environmental enrichment involves the conscious addition of environmental complexities to the rearing enclosures of fish species to mimic the natural habitat and improves the welfare of the farmed fish species (38–40). This complexity could be in the form of social, feeding, cognitive, structural or physical forms of enrichment (26). The physical form of enrichment involves the provision of structures like plants, sediments, stones, kelps, sand, gravels, artificial objects etc. in the rearing environment of captive fish to create a sensory and motor simulation that suites the behavioral and physiological needs of the fish (6, 41). Moreover, it offers the opportunity to use natural materials (plants, substratum) found within the fish species' habitat to improve their welfare without necessarily increasing the cost of production. *Eichhornia crassipes* (water hyacinth) are prevalent at the surface of many tropical and sub-tropical aquatic environments (42). The plant is available with a high proliferation rate and capacity to absorb nutrients in the tropical region (43). For instance, improved physico-chemical parameters of rearing water and higher growth rate were found in *Clarias gariepinus* reared in fish enclosures enriched with water hyacinth (43). In addition, Brunet et al. (41) found a positive welfare effect of the nature-based physical form of enrichment on farmed rainbow trouts. A reduced level of aggressive behavior was reported in two territorial fishes and *Tilapia rendalli* exposed to physical and structural enrichments (44, 45). Thus, the utilization of physical enrichment materials found in the natural habitat of *Clarias gariepinus*, which pose little or no financial implication, could be applied to enrich the rearing enclosures of this fish species for improved biological functioning and wellbeing.

Duncan (27) opined that the measurements of impaired biological functioning related to decreased health and increased physiological stress response could provide evidence that the welfare of an animal is compromised. Moreover, cortisol and glucose are the most commonly used indicators of the physiological response of teleost to stress (46, 47). Nevertheless, Broom (48) stated that cortisol provides no evidence of poor welfare because it has roles in positive and negative situations, which makes it erroneous to interpret its value as an indicator of poor welfare. However, variations in blood glucose levels are a primary stress response. It is a reliable biomarker to assess the hypothalamic-pituitary-adrenal (HPA) axis that indicates the condition and additional welfare benefits (49). The presence of stressors in the rearing enclosures of fish species considerably impacts their physiology, welfare and productivity. David et al. (50) reported using blood glucose and glycogen as indicators of stress response in freshwater fish species. Similarly, Endo and Wu (51) reviewed the use of blood glucose and cortisol as a good measure of assessing stress and fish welfare. Malini et al. (52) reported increased blood glucose as a physiological response in fishes exposed to environmental disturbance. In addition, Hossain and Beveridge (24) categorized the survival and growth rate trend in a fish rearing system as a crucial determinant of production success and an unambiguous indicator of animal wellbeing in a confined environment.

Behavioral indicators represent a generally non-invasive and early warning system of poor conditions in an aquatic environment (53). These behavioral tools include alteration in feed response, shoaling, aggression acts, swimming behavior etc., within the rearing enclosures of the fish (6). The rate of feed response in cultured fish species indicates the growth and production success of the farmer in the aquaculture sector (18, 54). Martins et al. (55) reported the relevance of shoaling behavior as a defensive behavior against predators and a good indicator of positive welfare in farmed fish species. In addition, Martins et al. (55) and Salvanes et al. (56) categorized changes in foraging behavior, aggression and group swimming of farmed fish species as an indication of acute and chronic stressors within the rearing environment. Moreover, the role of EE on behavioral traits of cultured fish species has been extensively documented (8, 9, 12, 38, 45). For instance, increased shoaling rate of cultured fish species reduced physical attacks, fights, food acquisition, and successful foraging behavior in zebrafish (56–59). Rosburg et al. (60) and Whiteet al. (61) verified the positive effect of environmental enrichment on the growth of chinook salmon, brown and rainbow trout. At the same time, Arechavala-Lopez et al. (39) and Zhang et al. (12) reported the positive influence of environmental enrichment on the growth, behavioral, physiological and welfare of *Sparus aurata* and *Sebastes schlegelii* in a laboratory environment.

However, there is a scarcity of information on studies related to the cumulative effect of environmental enrichment on *C. gariepinus* and its potential for application in the commercial production of this species. In this study, we hypothesized that all the provided forms of environmental enrichment during the 56-day culture period would improve the general well-being of *Clarias gariepinus*. Thus, the hypothesis of this study predicts that the provided forms of environmental enrichment would boost the survival rate and growth indices of the fish species while reducing the level of aggression and the blood glucose of the juveniles of the *Clarias gariepinus* under laboratory conditions.

## Materials and methods

### Study location

The research was carried out at the FUNAAB fish laboratory located between latitude 7^0^10'N and longitude 3^0^2'E.

### Experimental fish and acclimatization procedure

One hundred and eighty juveniles of *Clarias gariepinus* of 9-week-old were purchased from a private fish farm and transported in oxygen-filled polythene bags at 0700 h to the study site. The fish were acclimatized for 14 days in a rectangular fiber tank (6 x 4 x 3 m); they were fed twice daily (0900 h and 1,700 h) with Coppens feed (3 mm, Crude Protein = 45% and crude lipid = 12%, 4,300 kcal of digestible energy kg^−^1) at 3% body weight (16, 54).

### Experimental design and procedure

A total of 120 juveniles of active *C. gariepinus* with an average weight of 31.65 ± 0.69 g and a standard length of 11.2 ± 0.13 cm were selected from the purchased 180 catfish in the acclimatization tank. The fish were randomly stocked in 12 plastic tanks (1.7 × 1.2 × 1.0 m) of four treatments at 10 fish per tank in triplicates, representing an average weight of African catfish stocked per cubic meter (62). Each treatment was randomly exposed to plant enriched (PE), substratum enriched (SE), plant and substratum enriched (PSE), and barren/non-enriched (NE) tanks for a culture period of 56-days (11, 44). Each PE tank was filled with 10–12 stands of *Eichhornia crassipes*, popularly called water hyacinth; each stand contains 4–5 leaves with an average height of 15–20 cm above the water surface. The floating plants were collected from the outdoor fish enclosures located 10 m from the study site. The plants were washed with borehole water to get rid of snails and other likely pathogens; it was washed for 2 min under de-ionized water before placing them evenly at the surface of the plastic tanks (9). The SE tanks were filled with washed and sterilized fine sand substratum (grain size of 0.5–2.00 mm) to a depth of 1.5 cm in the culture tanks (8); PSE tanks were mixed at a ratio of one part of water hyacinth plant to one part of the fine sand substratum. The NE tanks were barren and plain, without any form of enrichment added to the tanks. The forms of enrichment used in this study are similar to what is obtainable in natural aquatic environments without any chemical interaction, which conforms with the specifications of Zhang et al. (12). Each tank was filled with water to two-thirds of its capacity with a flow-through system at a rate of 2.4 lhr^−1^. A weekly general tank cleaning and partial water exchange (9) were carried out throughout the study. The sides of the tanks were covered with opaque polythene material to reduce disturbance and interference during the study. All the 12 tanks were kept in the same laboratory room and exposed to the same photoperiod regime of 12L:12D. The water quality parameters were monitored with a multiparameter water probe (HANNA HI 98107 and HI 9143), and the mean values recorded during the experimental period for dissolved oxygen, temperature and pH were 6.5 ± 0.08 mg/l, 28.7 ± 1.0^0^C and 6.70 ± 0.51, respectively.

### Feeding pattern and growth indicators

The fish in each treatment and tank were fed with Coppens feed (3 mm, Crude Protein = 45% and crude lipid = 12%, 4 300 kcal of digestible energy kg^−^1) at a feeding rate of 3% per body weight. They were fed twice a day at 0900 h and 1,700 h (general feeding time) using the broadcasting method to ensure uniform access to feed by all the stocked fish. All uneaten feed (if any) was removed 30 min after feeding to prevent an alteration in the water quality in the culture tanks. Fish were weighed weekly to the nearest 0.01 g using Metler weighing balance (Model: 1,106) for an adjustment in the quantity of feed offered, while the standard length of fish was measured with a measuring board to the nearest 1.0 cm.

The initial body weight and weekly weight gain of the stocked *C. gariepinus* were recorded appropriately throughout the experimental period. The growth indicators (mean weight gain (MWG), specific growth rate (SGR) and feed conversion ratio (FCR) were evaluated for each of the treatments as follows:


(1) $$\begin{array}{l} Weight\;gain\;\left(g\right)=Final\;weight-initial\;weight. \end{array}$$

(2) $$\begin{array}{l} \begin{array}{l} Specific\;Growth\;Rate(g/day)= \end{array}\\ \begin{array}{l} {\left([\text{ln}(FW)-ln(IW)]/t\right)}^{*}100 \end{array} \end{array}$$

Where *ln* = Natural logarithm

*FW* = Final weight

*IW* = Initial weight

*t* = Duration of the experiment (in days) (63).


(3) $$\begin{array}{r} \begin{array}{r} Feed\;Conversion\;Ratio= \end{array}\\ Feed\;intake\;(g)/Bodyweight\;gain\;(g) \end{array}$$

(16).

### Survival rate

The survival rate (SR) of all the experimental African catfish was estimated using the equation illustrated below:


(4) $$\begin{array}{l} Survival\;rate\;=\;{\left[\left(INF\;-\;FNF\right)/INF\right]}^{*}100 \end{array}$$

Where *INF* = Initial number of fish stocked

*FNF* = Final number of fish stocked (16).

### Condition factor (k)

The condition factor (k) of the fish species was calculated to state their general wellbeing using Fulton's equation (64):

Condition factor (k)

$k=\frac{100W}{L3}$ .

Where *k* = Condition factor

*W* = Body weight(g)

*L* = Standard length (cm)

### Quantification of behavioral acts

Juveniles of *C. gariepinus* were observed at 08:00 h and 16:00 h, twice a week fortnightly, for 10 min per scan sampling using a focal sampling technique. The tanks were completely randomized for an appraisal at every observation time to eliminate bias. Each tank and treatment was observed for 120 min throughout the study period by two observers (with a timekeeper per observer). The sidewalls of the rearing tanks were covered with black polythene materials to prevent human disturbance during behavioral assessment (19, 54). The feeding response of the *C. gariepinus* in each treatment was assessed with a stopwatch. In addition, the frequency of aggressive acts displayed by the fish within the 10 min of observation time was counted and recorded in each tank per treatment. Furthermore, the duration of shoaling at the bottom of the tank as described by Miller and Gerlai (59) during the 10 min of observation by each treatment was recorded appropriately. The description of the behavioral traits measured in the study is given in Table 1.

**Table 1:** Ethogram of the measured behavioral variables.

**Behavioral traits**	**Description**
Feed response	Duration (in minutes) of time used by the fish to consume their given ration of feed
Aggressive acts	Frequency of instances of chasing that leads to contact between the mouth and body of a fish to inflict a mark or injury
Shoaling behavior	Duration (in seconds) of swimming together in clusters (six fish or more at ≤ 5 cm apart) at the lower one-third of the tank

### Blood sampling and measurement of blood glucose

Blood samples were collected fortnightly during the 56-day culture period to determine the physiological effect of the different forms of environmental enrichment on the stocked *C. gariepinus*. The blood samples were collected between 0700 and 0900 h. Sampled fish species (*n* = 3) per treatment were netted from the experimental tanks and anesthetized with MS222 in a 20 litres bucket of water; blood samples were collected at the caudal vein using a 2.5 ml heparinized syringe with 22G x 1½” according to the method of Di Marco et al. (65). Collected blood was gently pushed into a sterilized microfuge tube containing anticoagulant (20 mM EDTA). The whole blood withdrawal process took <5 min per fish to prevent discomfort. The samples were analyzed for blood glucose at the central Biotechnology Laboratory of the Federal University of Agriculture Abeokuta using the spectrophotometric method (47).

### Statistical analysis

All data obtained during the experiment were analyzed using the routines of IBM SPSS statistical packages (Version 23). The data were tested for normality using Shapiro-Wilk's test, while the homogeneity of data was tested using Levene's test. All the obtained data were not normally distributed and did not meet the assumption of ANOVA on normality and homogeneity even after transformation. The data were subjected to Kruskal-Wallis, a non-parametric test. Significant differences were reported at an alpha level of 0.05.

## Results

### Survival rate

**Figure 1:**
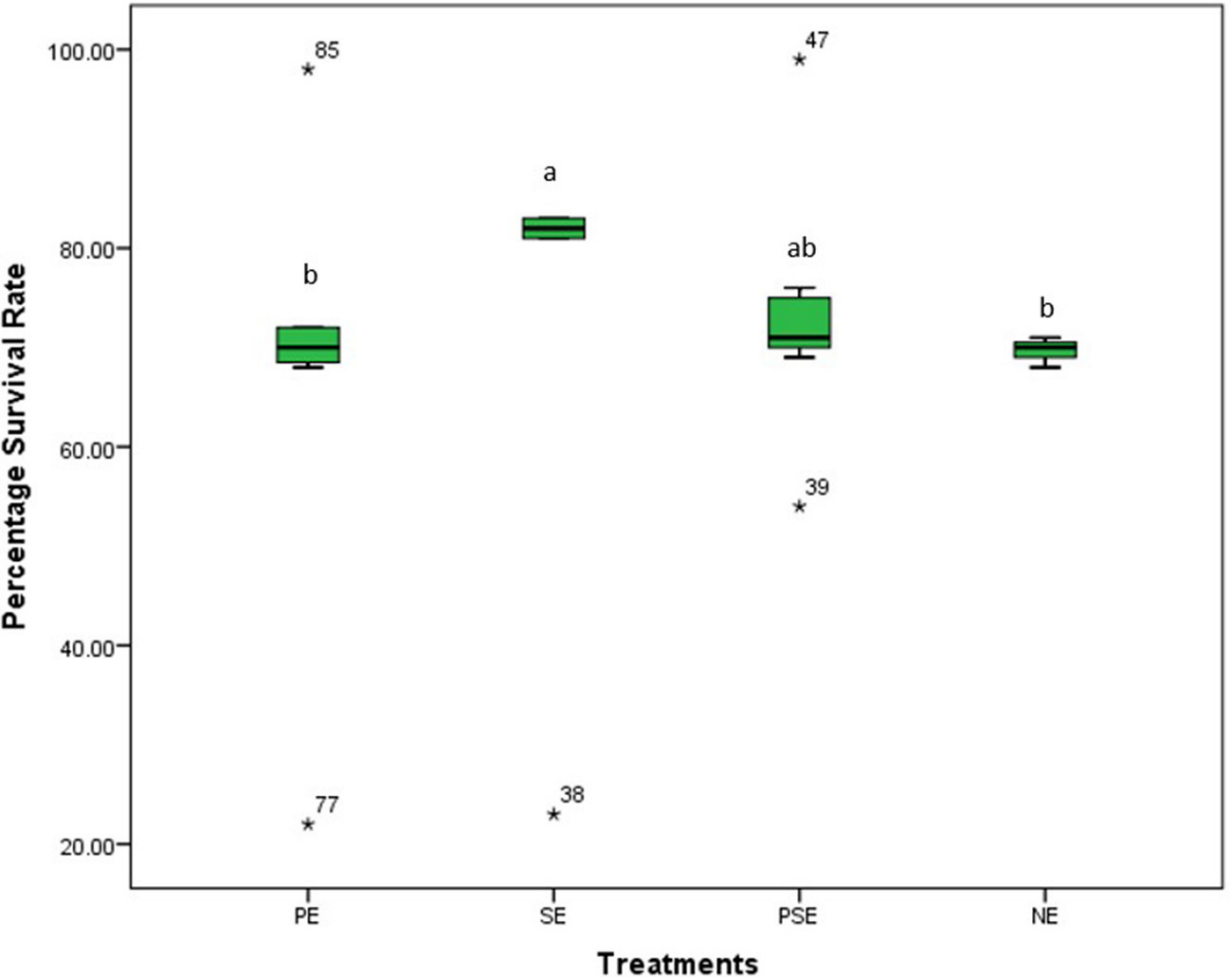
The survival rates of *Clarias gariepinus* exposed to plant enriched (PE), substratum enriched (SE), plant and substratum enriched (PSE) and non-enriched (NE) culture tanks. A significant difference between treatments was indicated with different letters at *p* < 0.05.

At the end of the 8 weeks study, there was a significant (χ^2^ = 77.31, df = 3, *p* = 0.01) difference in the survival rates of juveniles of *C. gariepinus* exposed to the different levels of environmental enrichment. The highest survival rate (83.4%) was recorded in SE, and there was no significant difference in the survival rates recorded in PE, PSE and NE throughout the study period (Figure 1).

### Growth indicators and condition factors of juveniles of *Clarias gariepinus*

**Figure 2:**
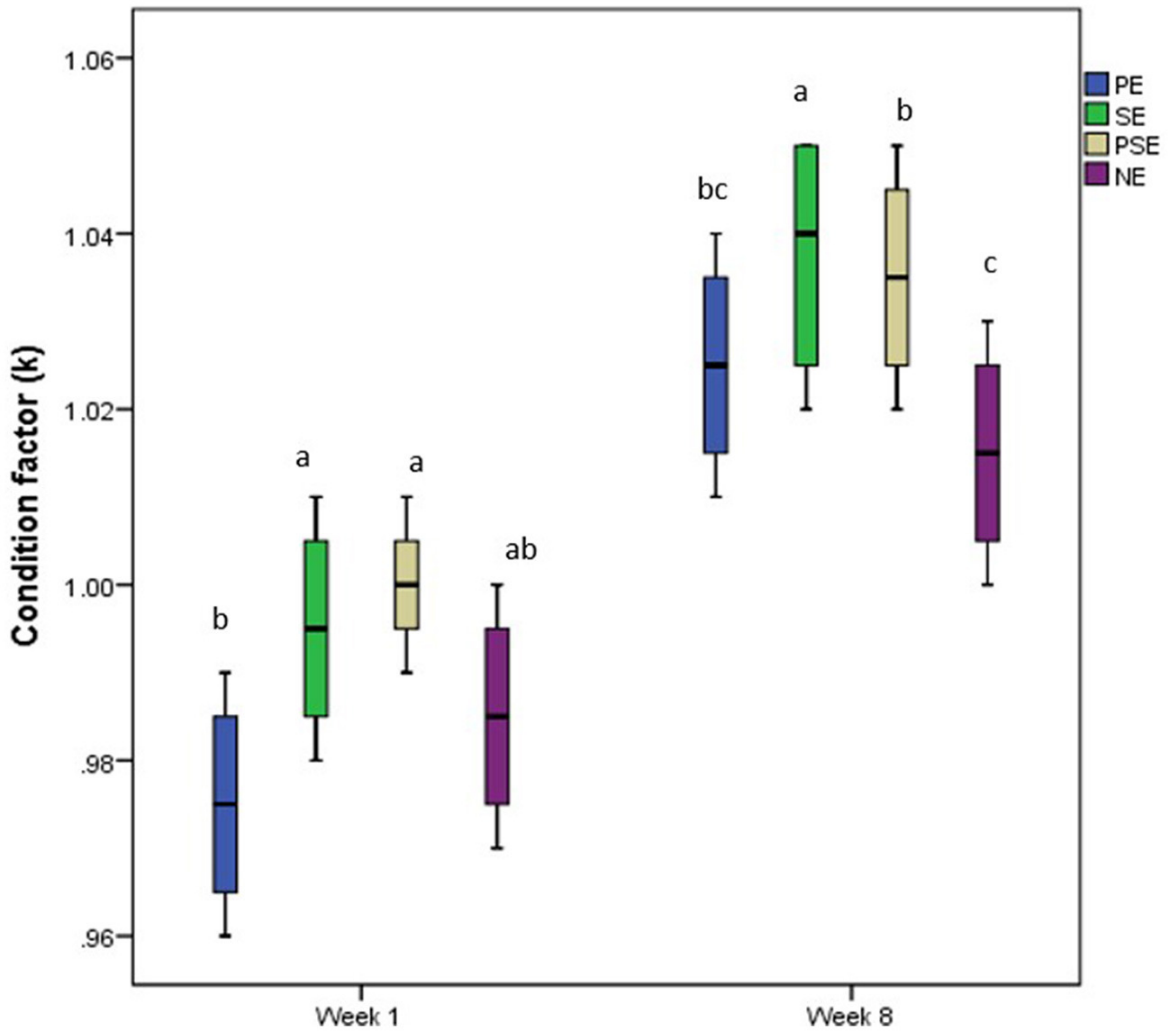
Change in condition factor (k) of juveniles of *Clarias gariepinus* exposed to different forms of environmental enrichment (PE, Plant enriched; SE, substratum enriched; PSE, plant and substratum enriched; NE, non-enriched) at week one and eight of the experimental period.

No significant (χ^2^ = 31.75, df = 3, *p* = 0.14) difference was observed between the initial body weight of *C. gariepinus* exposed to the different forms of environmental enrichment (Table 2). However, at the end of the culture period, a significant difference was observed in the final weight (χ^2^ = 90.51, df = 3, *P* = 0.02) and mean weight gain (χ^2^ = 58.87, df = 3, *P* = 0.04) of the cultured juveniles of *C. gariepinus* (Table 2). Fish reared with a substratum (SE) form of environmental enrichment had the highest mean weight gain (MWG) compared to the other forms of enrichment. The final body weight and MWG found in PE and NE were similar. In addition, the EEs had a significant (χ^2^ = 2.11, df = 3, *p* = 0.01) difference in the SGR of the cultured fish, but there was no significant difference between the SGR obtained at PSE and NE at the end of the culture period. Other estimated growth indices, such as FCR, were similar (χ^2^ = 1.04, df = 3, *p* = 0.10) between treatments after the 56 days of exposure (Table 2). Also, the condition factor of the *C. gariepinus* was similar at the beginning (week 1) and end (week 8) of the culture period. Still, a higher k-value was recorded in the fish exposed to SE enrichment (Figure 2).

**Table 2:** The growth parameters of *C. gariepinus* exposed to different forms of environmental enrichment under laboratory conditions.

**Growth indices**	**Plant enriched (PE.)**	**Substratum enriched (SE.)**	**Plant and substratum enriched (PSE)**	**Non-enriched (NE.)**
Initial body weight (g/fish)	31.64 ± 0.64^a^	31.63 ± 0.62^a^	31.64 ± 0.64^a^	31.68 ± 0.68^a^
Final body weight (g/fish)	88.83 ± 0.94^c^	93.52 ± 1.03^a^	91.37 ± 0.97^b^	88.33 ± 0.91^c^
Mean weight gain (g/fish)	57.19 ± 0.51^c^	61.89 ± 0.59^a^	59.73 ± 0.55^b^	56.65 ± 0.48^c^
SGR (%/day)	1.96 ± 0.11^c^	2.25 ± 0.17^a^	2.13 ± 0.12^b^	2.11 ± 0.09^b^
FCR	1.01 ± 0.06^a^^b^	1.09 ± 0.09^a^	1.06 ± 0.05^b^	1.01 ± 0.06^a^^b^

abc Mean values with different superscripts within a row are significantly (P < 0.05) different between treatments.

SGR, Specific Growth Rate; FCR, Feed Conversion Ratio.

### Behavioral traits of *C. gariepinus*

**Figure 3:**
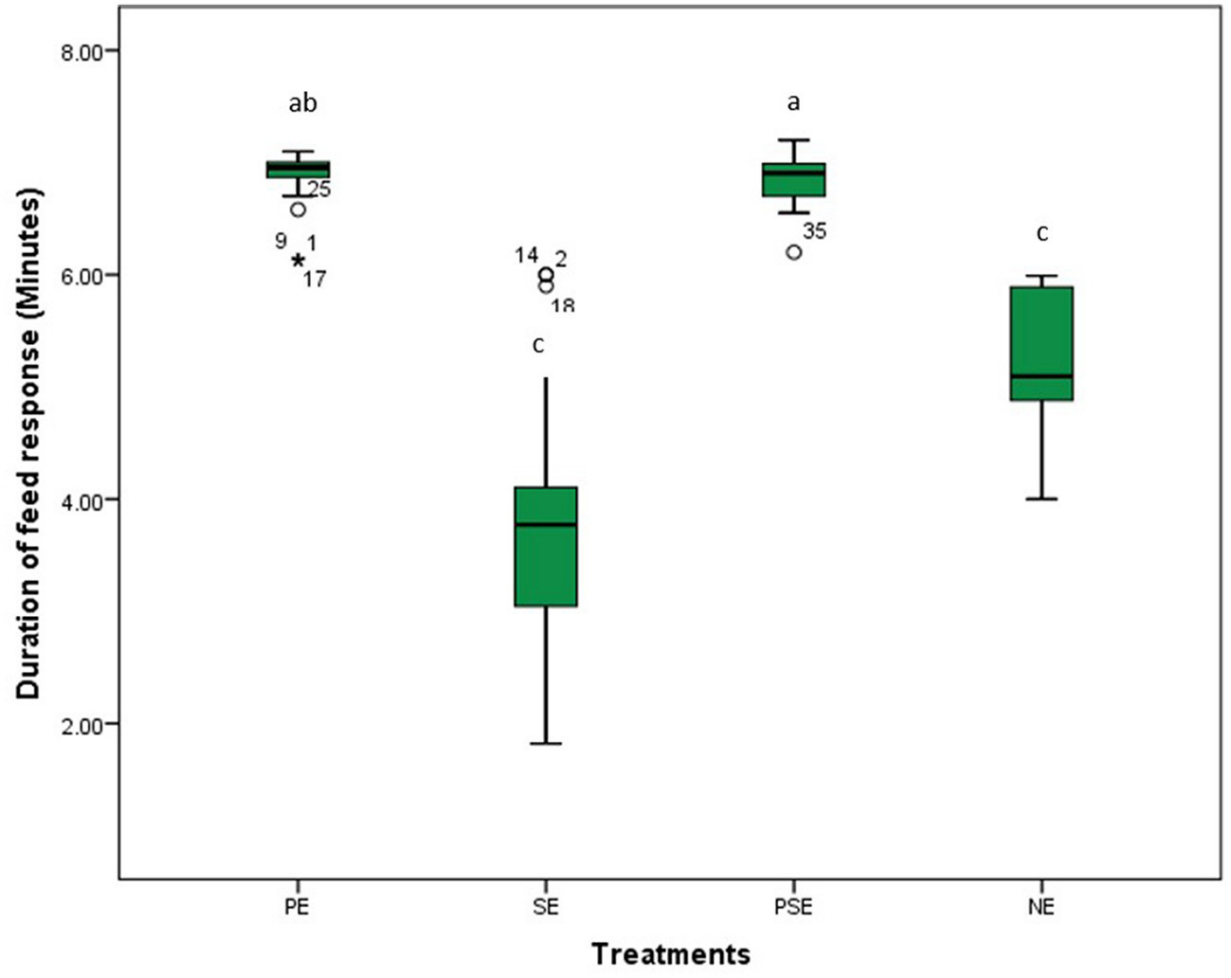
The effect of environmental enrichments on the feed response of juveniles of *Clarias gariepinus* under laboratory conditions. ^abcd^ Means of the duration of feed response differ (*p* < 0.05) between treatments.

**Figure 4:**
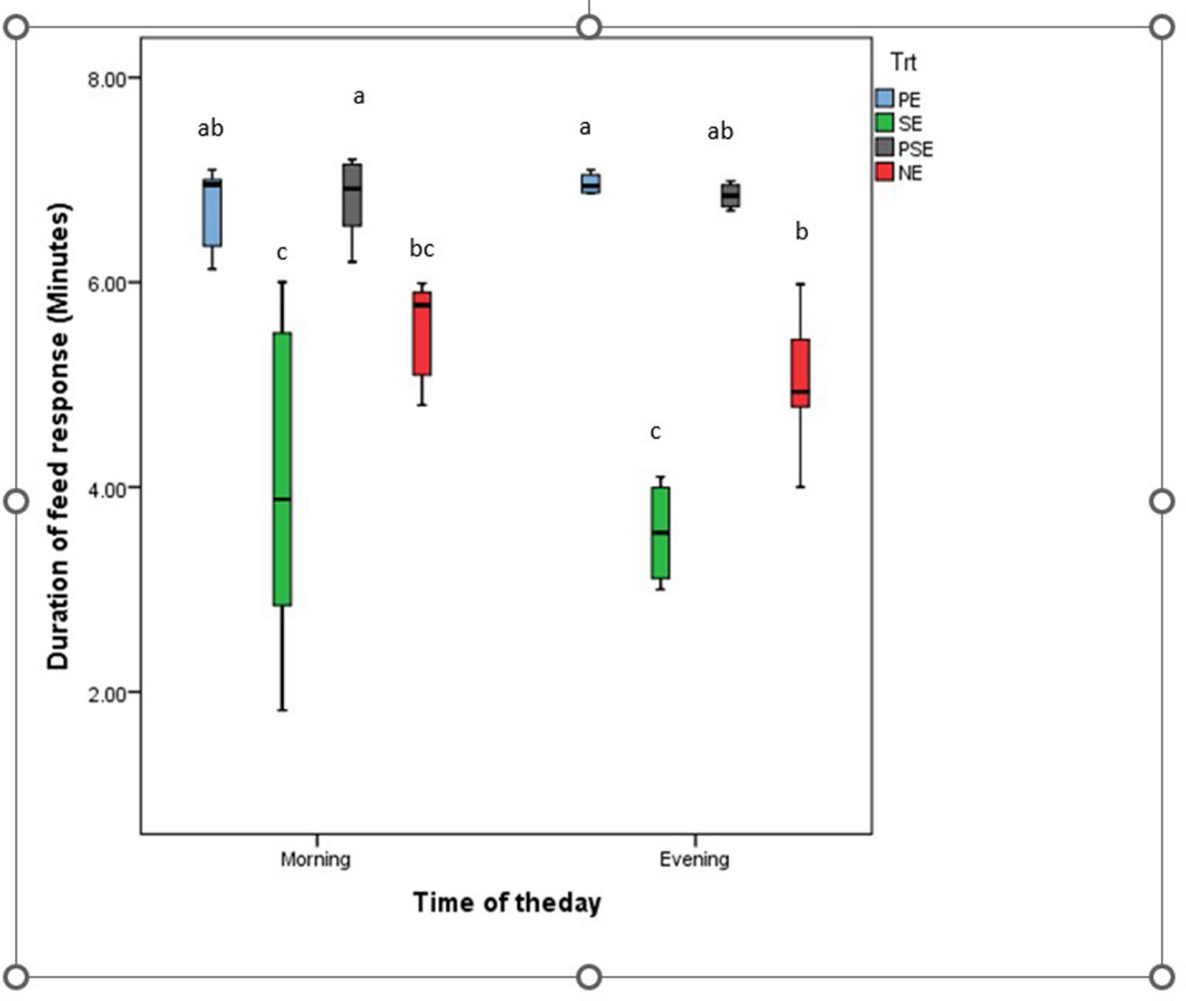
Average duration of feed response in juveniles of *Clarias gariepinus* exposed to different forms of environmental enrichment (PE, Plant enriched; SE, substratum enriched; PSE, plant and substratum enriched; NE, non-enriched) at two different times (morning and evening) of the day. Significant differences between treatments at each experimental period are marked with different superscripts at *p* < 0.05.

**Figure 5:**
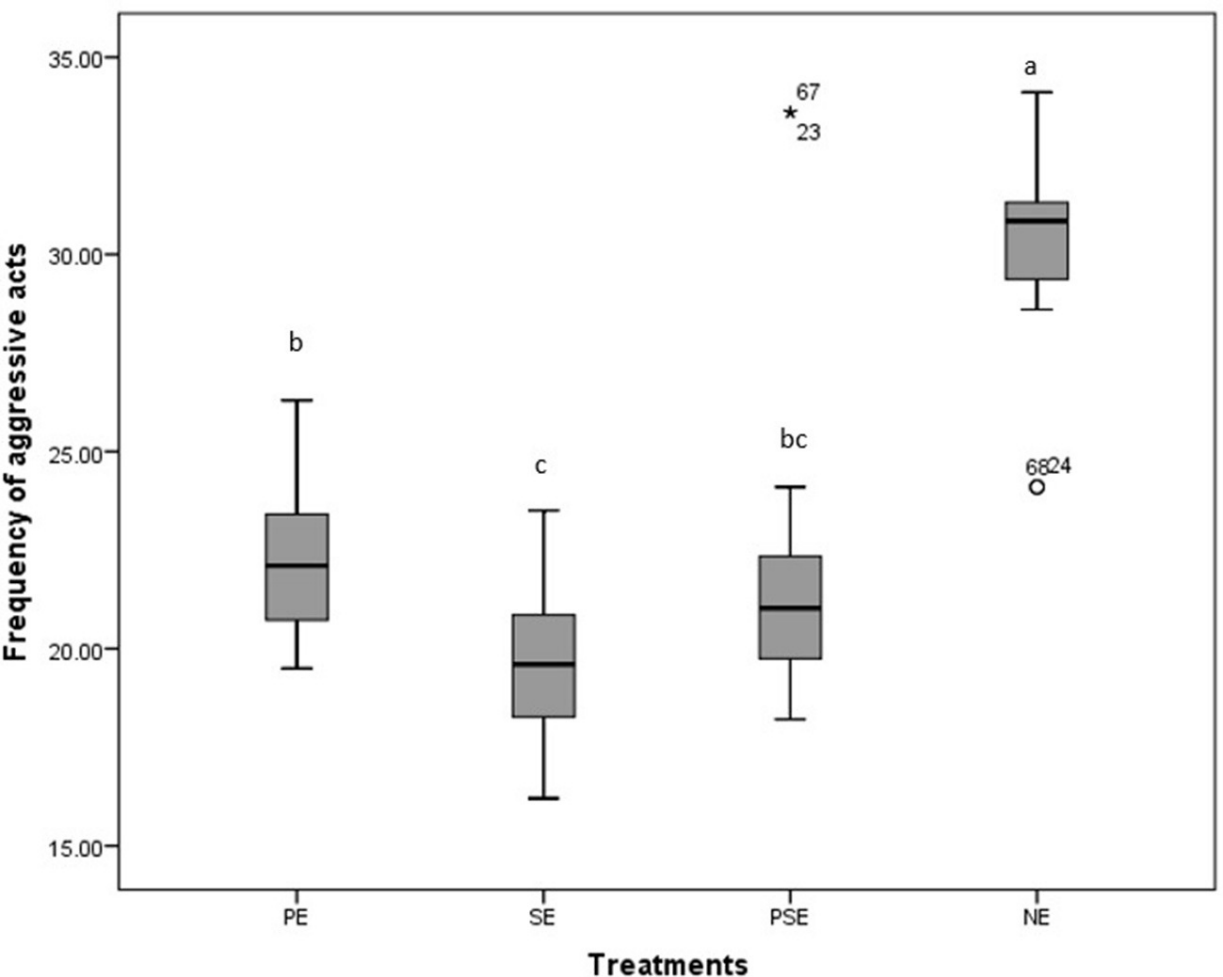
Boxplot of the effect of environmental enrichment during 600seconds on the frequency of aggressive acts displayed by juveniles of *Clarias gariepinus* under laboratory conditions. ^abcd^ Mean values with different superscripts were significantly (*p* < 0.05) different between treatments.

A significant difference was observed in the behavioral acts displayed by the juveniles of *C. gariepinus* exposed to the different forms of environmental enrichment throughout the study period. The fish in SE tanks took a shorter time to consume their diet. There was no significant difference (χ^2^ = 6.58, df = 3, *P* = 0.09) between the feed response of *C. gariepinus* exposed to PE and PSE forms of enrichment (Figure 3). In addition, the time of feeding during the day (morning and evening) had no significant effect on the feed response of the fish exposed to the different forms of environmental enrichment (Figure 4). Throughout the culture period, similar levels of aggressiveness were displayed by the juveniles of *C. gariepinus* exposed to PE (χ^2^ = 23.22, df = 3, *P* = 0.03), SE (χ^2^ = 19.93, df = 3, *P* = 0.04), and PSE (χ^2^ = 21.04, df = 3, *P* = 0.03) forms of enrichments. In addition, the highest (χ^2^ = 31.61, df = 3, *P* = 0.02) number of aggressive acts were displayed by fish reared in NE tanks compared to the other *C. gariepinus* cultured in other forms of EE treatments throughout the experimental period (Figure 5).

**Figure 6:**
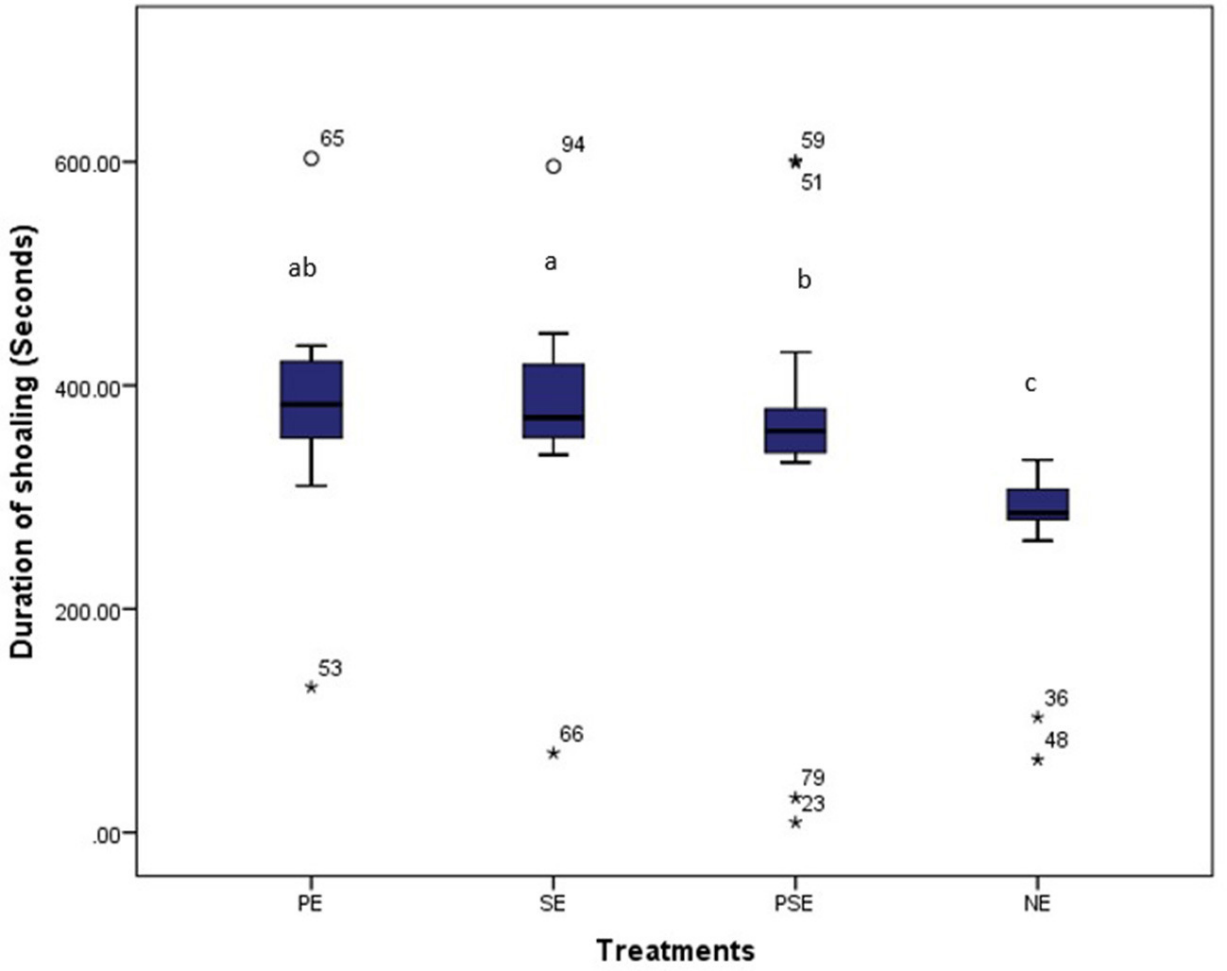
Average time (secs) spent by juveniles of *C. gariepinus* in displaying shoaling behaviour at plant enriched (PE), substrates enriched (SE), plant and substrates enriched (PSE) and non-enriched (NE) culture tanks. abcd Mean values with different superscripts were significantly (*p* < 0.05) different between treatments.

Also, the different EE treatments did not affect (χ^2^ = 391.42, df = 3, *P* = 0.10) the duration of shoaling displayed by *C. gariepinus*. There were similarities in the duration of shoaling behavior displayed by fish reared in PE (χ^2^ = 433.17, df = 3, *P* = 0.04), PSE (χ^2^ = 441.09, df = 3, *P* = 0.02) and SE (χ^2^ = 429.89, df = 3, *P* = 0.04) tanks. Besides, the least duration of shoaling within the period of observation was displayed by *C. gariepinus* reared in NE (barren) (χ^2^ = 283.09, df = 3, *P* = 0.02) tanks throughout the experimental period (Figure 6).

### Physiological response of juveniles of *C. gariepinus*

**Figure 7:**
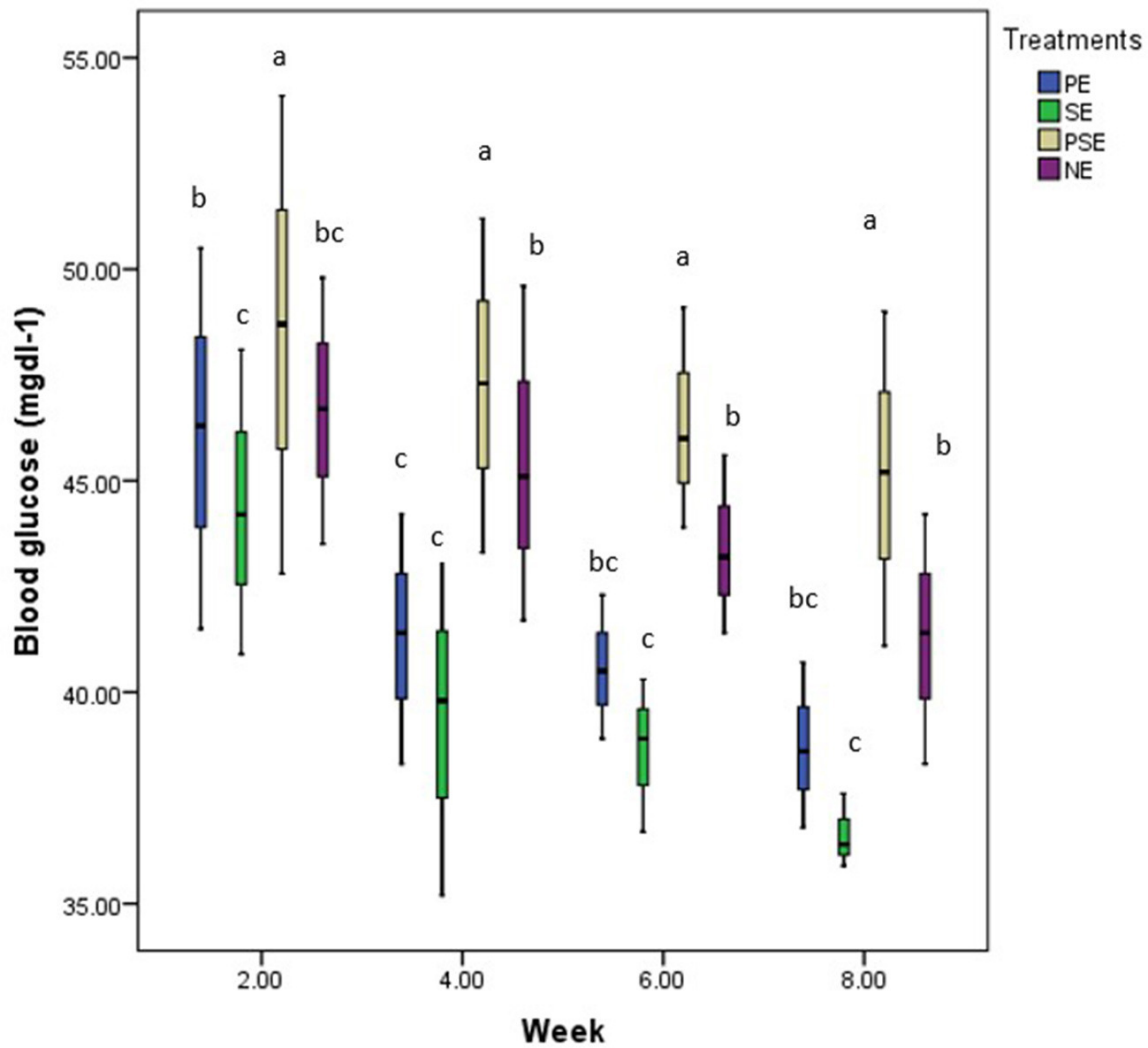
A boxplot showing the mean weekly glucose (mg/dl) values and bi-weekly trend of glucose in blood samples of *C. gariepinus* exposed to plant enriched (PE), substrates enriched (SE), plant and substrates enriched (PSE) and non-enriched (NE) culture tanks throughout the study period. ^abcd^ Mean values with different superscripts were significantly (*p* < 0.05) different between treatments.

The experiment consistently found the highest and least glucose values in PSE and SE tanks. At week two of the experiment, the EE treatments resulted in a slight increase in the glucose values obtained in the blood samples of *C. gariepinus* compared to the result obtained in week four across the treatments. By weeks six and eight, there was no significant difference in the glucose value recorded in all treatments. At the end of the experiment, the stress (glucose) level indicator showed that EE affected the level of glucose found in the blood of *C. gariepinus* during the study period (χ^2^ = 36.55, df = 3, *p* = 0.01) (Figure 7).

## Discussion

This study examined the survival rate, growth indices, condition factors, behavioral traits and physiological response of juveniles of *C. gariepinus* exposed to different levels of environmental enrichment for 56-days. The findings of this research accepted this study's hypothesis. The hypothesis' prediction that the provision of physical forms of enrichment would improve the growth and survival rate of the juveniles of *Clarias gariepinus* and lower the frequency of aggressive acts and glucose response in their blood samples was satisfied.

In our study, the survival rate of juveniles of *C. gariepinus* was affected by the different forms of environmental enrichment, with the highest and least survival rates in SE and NE tanks, respectively. This observed variance in survival rates could be due to physical structures such as natural aquatic plants and substratum that aid water quality in their rearing enclosures. The provided physical enrichments further serve as hiding structures to prevent physical attacks, cannibalism and subsequent mortality, which was absent in the non-enriched tanks with the least survival rate. This finding agrees with the result of Lee et al. (9) and Boerrigter et al. (66), who reported a high survival rate in juveniles of *D. rerio* and African catfish exposed to the physical form of enrichments. However, the result of this study contradicts the findings of Arechavala-Lopez et al. (39), who reported that the survival rate of juveniles of *Sparus aurata* was not affected by the structural form of enrichment.

Fish growth represents a complex physiological process often affected by feed intake, feed metabolism, feed conversion rate and the health status of the fish species. It can also be described as an indicator of the biological functioning of the fish species in its culture environment (17, 24, 67). Growth could increase, remain static or decrease depending on the severity of the wellbeing or condition of the fish in its rearing enclosure. The mean weight gain of the juveniles of *C. gariepinus* in this study was similar at the beginning of the experiment. However, the higher mean weight gain found in SE, PSE and PE compared to the NE tanks suggests good feed metabolism, feed conversion rate and wellbeing in their enriched rearing enclosure (16, 68). This result corroborates the findings of Zhang et al. (12), Batzina and Karakatsouli (69), and Rosengren et al. (70), who reported a higher growth rate in juveniles of black rockfish, gilthead seabream and Atlantic Salmon exposed to physical enrichments compared to those reared in barren tanks. In addition, the higher mean weight gain in the enriched (PE, SE, PSE) tanks might be due to the fact that the provided enrichments suites the basic needs of the African catfish compared to the fish exposed to the barren tanks (NE) (9). However, the result of this study contradicts the findings of Boerrigter et al. (66), who noted a decrease in the feed response and growth of African catfish exposed to structural enrichments (PVC-tubes), which was attributed to the high stocking density used in the study.

The similar condition factor reported in *C. gariepinus* at the different enrichment levels at the beginning and end of the culture period showed that the condition factor of the fish species exposed to enriched and barren tanks was not compromised throughout the experiment. Moreover, the higher k-value in African catfish exposed to the SE form of enrichment suggests that the provided form of enrichment met the requirements of the fish species in their rearing enclosures (18, 54).

This study found that *C. gariepinus* exposed to sediment enriched (SE) tanks took a shorter period to consume their diet. There were similarities in the duration used by African catfish exposed to PE and PSE to consume their feed ration. The observed similarities in the latency to feed displayed by fish in the SE and NE tanks compared to the PE and PSE tanks could be attributed to increased visibility which aids the zeal to feed and grow in the cultured fish species (40). This result is in line with the findings of Lee et al. (9) and Xu et al. (67) that reported an increase in the feed response and growth rate of zebrafish and rare minnows fish exposed to different forms of environmental enrichment. Moreover, the time of feeding during the day (morning vs. evening) had no significant effect on the feed response of *C. gariepinus* exposed to the different forms of environmental enrichment during the study (54). However, Zhang et al. (12) and Gregory and Wood (71) noted an inverse relationship between the presence of environmental enrichment and the feed response of juvenile Blackrock fish and rainbow trouts.

The environmental enrichments in this study affected the level of aggression displayed by the cultured juveniles of *C. gariepinus*. The least aggressive acts were recorded in SE tanks, probably because the fish shoals closer to the provided sediment than chasing or attacking each other within their rearing enclosure. This result corroborates the findings of Wilkes et al. (58), Boerrigter et al. (66), and Batzina and Karakatsouli (69), who reported decreased aggressive acts in *Danio rerio, C. gariepinus* and *Sparus aurata*. In addition, a relatively higher aggressive act was found in juveniles of *C. gariepinus* exposed to non-enriched tanks, which could be due to the barren nature of their rearing enclosure that aids visibility, frequency of encounter and the chances of establishing a territorial range within their tanks (44). This finding is similar to the result of Boerrigter et al. (66), who noted an increase in the aggression level of African catfish exposed to barren tanks in their study compared to the tanks enriched with PVC tubes. In addition, the frequency of aggressive acts was similar in PE and PSE tanks (40). Moreover, Arechavala-Lopez et al. (26) described environmental enrichment as a moderator of stress in fish by creating separate spaces to ease intraspecific aggression.

The observed higher shoaling rate displayed by juveniles of *C. gariepinus* close to the bottom of the water column or substratum found in fish exposed to SE tanks could be a defensive mechanism to discourage chases, unnecessary physical attacks, fights and injury to the fish. However, the duration of shoaling at the bottom of the tank enriched with PE, SE, and PSE did not vary throughout the culture period; this could be classified as an adaptive response for protection from predators (58). In addition, the similar duration of shoaling observed in these enriched tanks suggests an increased search for territory partners in their natural environment. This finding contradicts the result of Miller and Gerlai (59), who reported a decrease in the shoaling period of adult zebrafish exposed to physical forms of enrichment. However, the result of the present study agrees with the findings of Wilkes (57), who reported that zebrafish in enriched tanks shoaled more at the bottom of the tank compared to the same species reared in barren tanks.

Pankhurst (49) stated that poor conditions or impaired welfare in rearing enclosures are mostly accompanied by changes in the stress level of the fish. This stress level could be seen in blood parameters and other hormones, which might induce changes in a fish's survival, growth, behavior and physiology (51). The weekly trend of glucose levels found in the juveniles of *C. gariepinus* at the different forms of enrichment in this study further affirms the trend of aggressive acts displayed by the fish during the culture period. These findings implied that the SE form of enrichment is very beneficial for the welfare of *C. gariepinus* due to the reduced level of aggression and glucose recorded in the experiment. The consistent highest blood glucose over the culture period found in *C. gariepinus* exposed to PSE tanks could be a physiological process of adapting to and maintaining homeostasis in their internal environment.

## Conclusion

Modifying the rearing enclosures of juveniles of *C. gariepinus* greatly improved the survival rate, mean weight gain, condition factor, behavioral and physiological response of *C. gariepinus* under laboratory conditions. Environmental enrichment of the rearing enclosure of African catfish with fine sand substratum gave the highest mean weight gain and least aggressive traits. In addition, the highest stress (glucose) level was found in non-enriched (barren) tanks. The result of this study has a significant implication for improving the production efficiency of this important aquaculture species for fish food security and sustainability. Thus, modification of rearing enclosures for juveniles of African catfish with physical structures could be applied in commercial settings to simulate natural behavior, improve the growth rate, and reduce the aggressiveness of the fish species.

## Data availability statement

The original contributions presented in the study are included in the article/supplementary material, further inquiries can be directed to the corresponding author.

## Ethics statement

The animal study was reviewed and approved by Animal Ethics and Welfare Committee of the Federal University of Agriculture, Abeokuta.

## Author contributions

OO conceived the original idea, designed the methodology, wrote the manuscript, and supervised the execution of the project. MBO, CS, AB, and MO conducted the project. AA assisted with the blood collection and physiological analysis. SD did the statistical analyses. SD and IA proofread it. All authors contributed to the article and approved the submitted version.

## Conflict of interest

The authors declare that the research was conducted in the absence of any commercial or financial relationships that could be construed as a potential conflict of interest.

## Publisher's note

All claims expressed in this article are solely those of the authors and do not necessarily represent those of their affiliated organizations, or those of the publisher, the editors and the reviewers. Any product that may be evaluated in this article, or claim that may be made by its manufacturer, is not guaranteed or endorsed by the publisher.
